# DNA Repair in Cancer

**DOI:** 10.1155/2019/8676947

**Published:** 2019-07-24

**Authors:** Zhihua Kang, Qingyuan Yang, Yintao Li

**Affiliations:** ^1^Department of Laboratory Medicine, Huashan Hospital, Fudan University, Shanghai, China; ^2^Department of Radiation Oncology, Rutgers Cancer Institute of New Jersey, Rutgers University, New Brunswick, NJ, USA; ^3^Department of Radiation Oncology, Massachusetts General Hospital, Harvard University, Boston, MA, USA; ^4^Molecular and Cellular Oncology, MD Anderson, The University of Texas, Houston, TX, USA

DNA repair system has evolved to maintain the genomic integrity to defend against both endogenous and exogenous sources of DNA damage, such as endogenous factors include reactive oxygen species, replication errors or mistakes in meiosis and exogenous factors include ultraviolet (UV) radiation, ionizing radiation (IR), and some other chemicals or chemotherapeutic agents. Multiple repair pathways (direct repair, base excision repair, nucleotide excision repair, mismatch repair, nonhomologous end joining, and homologous recombination pathways) can be aroused from the diverse forms of DNA lesions including mismatch paired bases, small deletions or insertions, and DNA single or double-strand breaks. These repair pathways also exert crosstalk with others to complete the whole DNA repair process.

The deficient DNA repair causing prolonged existence of DNA damages can lead to genes mutations, chromosome rearrangements, genomic instability, and finally carcinogenesis. Indeed, defects in DNA repair pathways contribute to many heritable cancer predisposition syndromes; however, cancer-related DNA repair deficiency may also occur in sporadic cancer case. Defective DNA repair is common in carcinogenesis and plays a critical role in cancer progression. For example, genetic mutations in DNA mismatch repair genes are involved in reducing mismatch repair and increasing the risk to colon and uterine tumors; BRCA1, BRCA2, and PALB2 genes mutations result in defective homologous recombination repair and are associated with the carcinogenesis of breast and ovarian cancer. In these years, many cancer-related germline mutations in DNA repair have been reported; thus to detect these genetic variations gives us a chance to evaluate the cancer risk of the individual with these mutations.

Furthermore, these defects in DNA repair pathways may have therapeutic implications for clinical practice. Most recently, a series of therapeutic strategy have been exploited, such as platinum chemotherapies and PARP inhibitors in homologous recombination defected breast and ovarian cancers and inhibitors of immune checkpoints CTLA-4, PD1/PD-L1 in the case of mismatch repair deficiency cancers. These observations give the direction for further research to investigate the defects in DNA repair pathways that may serve as very useful biomarkers for the choice of suitable oncotherapy.

This special issue includes 4 high-quality peer-reviewed articles and 1 review that brings us new ideas and findings in DNA repair in different types of cancers focusing on the genetic changes in DNA repair genes, the influences from these changes to carcinogenesis, and also the therapeutic implications. We have reasons to believe that these articles will enlighten and motivate not only the new inspirations but also the scientific advances in the study of DNA repair in cancer.

